# Supplementary material for: Expression of Specific Alleles of Zinc-Finger Transcription Factors, HvSAP8 and HvSAP16, and Corresponding SNP Markers, Are Associated with Drought Tolerance in Barley Populations
Source: Int J Mol Sci. 2021 Nov 10;22(22):12156. doi: 10.3390/ijms222212156 (PMC8617764; doi:10.3390/ijms222212156)
Supplement: Supplementary file 1 [file ijms-22-12156-s001.zip › ijms-1446968-supplementary.pdf]

International Journal of
Molecular Sciences

MDPI

Supplementary Materials

Expression of specific alleles of Zinc-finger transcription factors, *HvSAP8* and *HvSAP16*, and corresponding SNP markers, are associated with drought tolerance in barley populations. Baidyussen, A.; Jatayev, S.; Khassanova, G.; Amantayev, B.; Sereda, G.; Sereda, S.; Gupta, N.K.; Gupta, S.; Schramm, C.; Anderson, P.; Jenkins, C.L.D.; Soole, K.L.; Langridge, P.; Shavrukov, Y.

Supplementary Material S1. Published information about expression analysis of *HvSAP8* and *HvSAP16* homologs in monocot and dicot plant species in dehydration, salinity and other abiotic stresses.

**Species and references**	**Homologs to *HvSAP8* (A20-AN1)**			**Homologs to *HvSAP16* (AN1-C2H2)**		
	**Dehydration**	**NaCl**	**Other**	**Dehydration**	**NaCl**	**Other**
			**Monocot plant species**			
Rice (*Oryza sativa*) indica, cv. Pusa Basmati 1 [4]	*OsSAP8:* Upregulated: 3.5-fold, (air-dry, 6 hrs)	Upregulated: 3-fold (200 mM NaCl)	Cold. Non changed: =1.	*OsSAP16* =2-fold	=1.5-fold	Cold. Non changed: =1
Rice (*Oryza sativa*) indica, cv. Jiucaiqing [10]	ZFP184 = *OsSAP8* = 2-fold (20% PEG 6000), 12 hrs		Cold Non changed: = 1			
Rice (*Oryza sativa*) indica, cv. IR-50, 8-days-old [11]. Improve tolerance in transgenic tobacco and rice	*OsSAP8:* Strongly expressed (both 400 mM manutol and air-dry)	Strongly expressed (250 mM NaCl)	Cold, heat, submerge, wounding – all strongly expressed			
Rice (*Oryza sativa*) indica, cv. IR36, shoots, 14-days-old [12]	*OsSAP8:* Up to 2.6-fold	Up to 1.5-fold				
Bread wheat (*Triticum aestivum* L.) [17]				*TaSAP17 (=OsSAP16).* Fluctuated up to 1.2 and down up to 0.5 around 1	Up-regulated to 4- and 6-fold (leaves and roots) in salt tolerant cvs. Down regulated to 0.1 and 0.3 (leaves and roots) in salt sensitive cvs.	
Tall fescue (*Festuca arundinacea*) [13]	*FaZnF* (=*OsSAP8*): Highly expressed at 12-24 hrs	Gradually increased and most	Heat (40°C) – UV – wounding (12			

	(PEG6000 = 12.87%). Most highly expressed at 24-48 hrs (drought)	highly expressed at 24 hrs (500 mM NaCl)	hrs) Highly expressed at 2/1hrs-24 hrs			
Foxtail millet (*Setaria italica* L.), cv. Yugu 1 [14]	*SiSAP4* (similar to <u>*OsSAP4*</u>): Up-regulated to 4-fold at 1-3 hrs and gradually returned back to 1-1.5 at 12-24 hrs. (6% PEG-6000),	Up-regulated to 1.5-fold at 9 hrs and returned back to 0.8-1.2 at 12-24 hrs. (100 mM NaCl)	Cold. Two small peaks (1.5-fold) at 0.5 and 12 hrs			
Banana (*Musa acuminate*) cv. Karibale Monthan [16]	*MusaSAP1* = (*HvSAP8*) Up=2.0 (5, 15 hrs); down=1.4 (24 hrs) Drought	Up=2.2 (5 hrs); down=2 and 1.7 (15 and 24 hrs) (250 mM NaCl))	Up=2-3, cold; Up=3-10, heat; Up=1.8-2.2, wounding			
Sugarcane (*Saccharum officinarum* L.) cv. 'Badila'. Overexpression in transgenic tobacco [15]	*ShSAP1* = Up-regulated until 2-fold at 10 and 15 days	Similar to: *OsSAP8*. 1.8-2.2-fold (200-400 mM NaCl)				
**Dicot plant species**						
Rapeseed (*Brassica napus*) [18]	*BnaA06g02460D* (= *AtSAP2* = *HvSAP8*). Not changed = 0	Not changed = 0	Heat: Very high; Cold: Not changed = 0	*BnaA0936780D* *BnaC08g28470D* (= *AtSAP13* = *HvSAP16*) Not changed = 0	Not changed = 0	Heat and cold: Not changed = 0
Cucumber (*Cucumis sativus* var. *sativus*), line 9930 [45]	*CsSAP11* – closest to *AtSAP2* (High expression in all non-stressed tissues)			*CsSAP6* – closest to *AtSAP11:* 6-fold increase until 12 hrs and drop to 1.5-fold at 24 hrs. 10% PEG-6000)	Gradually increase to 8-fold until end (24 hrs). 200 mM NaCl	Cold: 2.7-fold increase until 12 hrs and steady
Tomato *(Solanum lycopersicum)* [46]	All *Sl* genes with specific *SlSAP2, 3, 4* and *5* (all closest homologs to *OsSAP8*). Up=1.8	Up=5	Cold-Heat: Up=1.5; down=0.6; Wound-Submergence: Down=0.5; up=1.5	*SlSAP12* – closest homolog to *AtSAP11* Up=1.2	Up=1.7	Cold-Heat: Down=0.6 and 0.7; Wound-Submergence: Up=1.3&1.5

Castor bean (*Ricinus communis*) [21]	*RcSAP4* – closest to *AtSAP2:* Down to 0.5 (3 hrs), up to 1.5 (6 hrs) and down again (12 hrs) 20% PEG-6000)	Gradually up to 1.5 (300 mM NaCl)	Heat, 50°C: Gradually up to 7 (6 hrs) and return (12 hrs); Cold: Gradually down to 0.5	*RcSAP9* – closest homolog to *AtSAP11.* Strongly down to 0.1-0.2	Up to 14 and down to 0.5	Heat, 50C: Up to 1.6 in the middle; Cold: Gradually up to 7
Medicago truncatula [22]	*MtSAP4, -13* – closest homolog to *HvSAP8*- Gradually up to 6 and 3 (12 hrs) (whole seedlings on paper towel)	Gradually down to 0.5 (12 hrs) (300 mM NaCl)	Cold: No changes and slightly up to 2 (12 hrs)	*MtSAP16; -17* – closest homolog to *HvSAP16* - No changes	No changes	Cold - No changes
Soybean (*Glycine max*) [47]	*GmSAP10; -13; -18; -19* - all closest homologs to *OsSAP8*: Up gradually to 10-, 13-, 13-, 4.5-fold (at 24 hrs) (whole plants on paper towel)	Up gradually to 2.2, 1.4, 2.5, 3.2 (at 8-12 hrs) (250 mM NaCl)		*GmSAP1; -2; -9* - all closest homologs to *OsSAP16*: Up gradually to 9-, 22-, 7-fold (at 8-12 hrs)	Up gradually to 4, 2.5, 5 (at 8-12 hrs)	
Cotton, tetraploid (*Gossypium hirsutum*) [19]	*GhSAP1; -12; -16* - all closest homologs to *OsSAP8*: Up to 2-, 0-, 2-fold. Heatmap: All three little up at 1 h and down after (PEG, 20%)	Up to 5-, 2-, 2-fold. Heatmap: All three strongly up at 1 h and down after (300 mM NaCl)		*GhSAP11* - closest homologs to *OsSAP16*: Down, 5-fold. Heatmap: neutral, little down (6 hrs) and little (12 hrs)	Up to 4-fold. Heatmap: Strong up at 1 and 12 hrs; and moderate at 3 and 6 hrs	
Peach (*Prunus persica*), non-dormancy buds, two cultivars, budsticks cut in water [48]	*PpSAP1* = *OsSAP4* and *OsSAP8* = Significantly increased to 1.4-1.6-fold in Day1 and Day 3 (removed water)	Significantly increased to 1.2-1.5-fold in Day1 and Day 3 (200 mM NaCl)				
Kashgar tamarisk (*Tamarix hispida*), woody halophyte, (Log_2 scale) [20]	*ThSAP2; -10* - closest homologs to *OsSAP8* – Up to 2 (leaves) and mixed -2 - +3 (roots) (20% PEG)	Up to 2 (leaves) and mixed -0.5 - +1 (roots) (150 mM NaCl)		*ThSAP7* - closest homologs to *OsSAP16* – Up to 1 (leaves) and mixed -0.5 - +2 (roots)	weak up 1 (leaves), weak down -1 (roots)	

International Journal of *Molecular Sciences*

MDPI

Supplementary Material S2. Annotated sequence accessions of Morex, Barke and Bowman in databases with the promoter regions of *HvSAP8* and *HvSAP16* genes. Primers designed and used for sequences are highlighted in blue. Start codon of the coding regions are shown in green. The identified SNPs in the studied barley germplasms are designated in red.

HvSAP8

>Morex_contig_273714 CAJW010273714 carma=7HL POPSEQ: chr=7H cM=85.9773371104815 (Promoter area = 1.180 bp)

CCACACCCACGGCGGGGACGGCCGCAGAATGAGAGAAAAAAAATATCGGCGCGGGGCCCCTGGAGGGGCCCCCCCGTCATTCCCCCG
CTGCCCCCC**TCTCCTCCCTTTTATCCCTCC**GCCCACCTCCCCACGTTTCGCCCCCATTTCGAAGCCCACCGAATTCCTCGCGATTCC
TCTCCCCTCGCCTCCTCGTCCTCCCCCTCCTAGGGTTAGTACCCCCTCCCCTCCCCTCCCCCCGGATTCCACCGCGGGTCGACCTCT
CCCCCCGGATCCGCCTCCAGATTTAGGGCTCCGGCTCCGCCGATCCGGGCTCGTTCTTTTCCGCCGGAGCTTTTCCGTCGCTGATGA
TTTCTTGTTGTTGGGTCCGTGTGCAGGGATCGCCGGAGAGGAATCGCGACGAGGGCTTCCTCCTATCAGGTTCGTGGCTCGCCGACC
CTCCGTTCCCGGTGATAATCATCTGTCTTCCCTAGGAAGTTCTGCCATAGCCTCTGTGCTTTTATTTATTCCTCTTGGTCAAAAATC
CCTGATTTTCCTCTGGCCGTCGATTCCTCCGGCCGCACCAGAAGCCACCGGCCGGGTACCGATTTGTTGGCTCGACGTTCAATTCGG
CAGAACCGCGACGAGCCACGGCCAAATTCGGCGCTCCGATGCAGTAAAGGCGGCCGGCCTCCTTTACACGTATCCGTCCGGTGTGTT
TGTTGCCGCGGCCGGTTGTTGATGCGGCCGCTTTCGCAAAAGTTAGTGCGCGGATACGGTTTGCTACTTTACGGATTTGTTGCCGCT
GTTGCGTAAAGCGGCGTAAAGTCGGGGCCTTTCTTTAGAATTAAATTTCCCCTGCTCTGCTCCGATGCCGGTAGGTGATTAGGCCGC
TGTTGGCTAATTATAAGCCAGGCAGGTTGCATCGTTTAGTGCTGGTGCCTTCAGCTCTGTGTTTGACCAAGCAGCCACCACTATCCT
TTTGTAAGATGCTTCGTAGAGCACCTTGCTTTACCAGAGTTGGATTCAGTAGGCTAAAACATACTGTATGTTTGAAGATTATGCCAT
GTTCATCTCTAAATTGTTAAAGCCGCAGTGATTGTTCGAAAATACTTACAGTTTGTGCCTTGCTGTTTATCATAGCCCTTGTGGGTT
TTGTTGTGTT**GACCGTGTTGTGTCTGGTTTGTA**GTTAATTAACCAAAGCCATGGAGCACAAGGAGACGGGCTGCCAGTCACGGGAGG
GTCCGATCCTTTGCGTCAATAACTGCGGCTTCTTTGGCAGCG

>Bowman_contig_10544 CAJX010009841 carma=7HL (Promoter area = 1,180 bp)

CCACACCCACGGCGGGGACGGCCGCAGAATGAGAGAAAAAAAATATCGGCGCGGGGCCCCTGGAGGGGCCCCCCCGTCATTCCCCCG
CTGCCCCCC**TCTCCTCCCTTTTATCCCTCC**GCCCACCTCCCCACGTTTCGCCCCCATTTCGAAGCCCACCGAATTCCTCGCGATTCC
TCTCCCCTCGCCTCCTCGTCCTCCCCCTCCTAGGGTTAGTACCCCCTCCCCTCCCCTCCCCCCGGATTCCACCGCGGGTCGACCTCT
CCCCCCGGATCCGCCTCCAGATTTAGGGCTCCGGCTCCGCCGATCCGGGCTCGTTCTTTTCCGCCGGAGCTTTTCCGTCGCTGATGA
TTTCTTGTTGTTGGGTCCGTGTGCAGGGATCGCCGGAGAGGAATCGCGACGAGGGCTTCCTC**T**TATCAGGTTCGTGGCTCGCCGACC
CTCCGTTCCCGGTGATAATCATCTGTCTTCCCTAGGAAGTTCTGCCATAGCCTCT**C**TGCTTTTATTTATTCCTC**C**TGGTCAAAAATC
CCTGATTTTCCTCTGGCCGTCGATTCCTCCGGCCGCACCAGAAGCC**G**CCGGCCGGGTACCGATTTGTTGGCTCGACGTTCAATTCGG
CAGAACCGCGACGAGCCACGGCCAAATTCGGCGCTCCGATGCAGTAAAGGCGGCCGGCCTCCTTTACACGTATCCGTCCGGTGTGTT
TGTTGCCGCGGCCGGTTGTTGATGCGGCCGCTTTCGCAAAAGTTAGTGCGCGGATACGGTTTGCTACTTTACGGATTTGTTGCCGCT
GTTGCGTAAAGCGGCGTAAAGTCGGGGCCTTTCTTTAGAATTAAATTTCCCCTGCTCTGCTCCGATGCCGGTAGGTGATTAGGCCGC
TGTTGGCTAATTATAAGCCAGGCAGGTTGCATCGTTTAGTGCTGGTGCCTTCAGCTCTGTGTTTGACCAAGCAGCCACCACTATCCT
TTTGTAAGATGCTT**T**GTAGAGCACCTTGCTTTACCAGAGTTGGATTCAGTAGGCTAAAACATACTGT**G**TGTTTGAAGATTATGCCAT
GTTCATCTCTAAATTGTTAAAGCCGCAGTGATTGTTCGAAAATACTTACAGTTTGTGCCTTGCTGTTTATCATAGCCCTTGTGGGTT
TTGTTGTGTT**GACCGTGTTGTGTCTGGTTTGTA**GTTAATTAACCAAAGCCATGGAGCACAAGGAGACGGGCTGCCAGTCACGGGAGG
GTCCGATCCTTTGCGTCAATAACTGCGGCTTCTTTGGCAGCG

HvSAP16

>Bowman_contig_183489 CAJX010179134 carma=2HS (Promoter area = 790 bp)

CCCCCCCCCCCCCCTCGATTCTC**ATCCCAACATGCAGTCCCTACA**TATATTAATCTGGCGATGCTTACCTGAAGTATCTGTTTAAAA
ACTATAAAGTGTGGCAGAATTTTGAATACTGACTAAATATAAGAGATGCAAAATCCTTTCTTTTCGCTCAAAATGTATTCAATCCTT
CTGGACTTCCTGACTTTCTTAATATGAAAATAAGATTGCACATTTGTACTGAGGATGGTAGTTATTTATAGCGATTAGTAAAGATTG
CACGCGATTAG**A**GAAGCTTGTCCAACATCGTCTGANNNNNTTTGGGCATATTCAGCGCAGGCCTCCAGAAGCCCCAGTGCATAGCGG
ACGGCTAAAGCATTGTTGATAATGTCAAGAGAGGCCAGGGTAGACCGAATTTGACATGAGAAGAGTCCGTAAAGAGAGATCTAAAGG
ATTGGAGCATCACCAAAGAACTAGCCATGGACTGGGCTGCGTGGAAGCTTGCTATCCATGTGCCAGAACCATGAGTTGGTCGCGAGA
TCTAATGGGTTTCACCTCTAGCCTACCCNNNNNNNNNNNNNNNNTAAAGGCTTTGTTGTTGTTGTTGTGGTAGTGAAGAGTGAAGTTT
TTTGCTGAGTTAACTGTATTCAACTTTTTTTTCACAGAATATGTAGTATTTTATTCTTATGGAAATTGATTCCATTTTACCTTCTGA
AACTATTTTGTCTTTTGAAATGCACTGGCACCTTAATTATAATAGTTCTGTCATCACCAAG**TGCAGCCAGCCATTGTGCTAAT**ATGG
CTATTGTATGATTGTTCAGGTCTTTTGCCTTCAGCACCGAAGTTATACATCACATAA

>Barke_contig_2792434 CAJV012605985 carma=2HS (Promoter area = 790 bp)

CCCCCCCCCCCCCCTCGATTCTC**ATCCCAACATGCAGTCCCTACA**TATATTAATCTGGCGATGCTTACCTGAAGTATCTGTTTAAAA
ACTATAAAGTGTGGCAGAATTTTGAATACTGACTAAATATAAGAGATGCAAAATCCTTTCTTTTCGCTCAAAATGTATTCAATCCTT
CTGGACTTCCTGACTTTCTTAATATGAAAATAAGATTGCACATTTGTACTGAGGATGGTAGTTATTTATAGCGATTAGTAAAGATTG
CACGCGATTAG**T**GAAGCTTGTCCAACATCGTCTGAGATGGTTTGGGCATATTCAGCGCAGGCCTCCAGAAGCCCCAGTGCATAGCGG
ACGGCTAAAGCATTGTTGATAATGTCAAGAGAGGCCAGGGTAGACCGAATTTGACATGAGAAGAGTCCGTAAAGAGAGATCTAAAGG
ATTGGAGCATCACCAAAGAACTAGCCATGGACTGGGCTGCGTGGAAGCTTGCTATCCATGTGNNNNAACCATGAGTTGGTCGCGAGA
TCTAATGGGTTTCACCTCTAGCCTACCCNNNNNNNNNNNNNNNNNNAAAGGCTTTGTTGTTGTTGTTGTGGTAGTGAAGAGTGAAGTTT
TTTGCTGAGTTAACTGTATTCAACTTTTTTTTCACAGAATATGTAGTATTTTATTCTTATGGAAATTGATTCCATTTTACCTTCTGA
AACTATTTTGTCTTTTGAAATGCACTGGCACCTTAATTATAATAGTTCTGTCATCACCAAGTGCAGCCAGCCATTGTGCTAATATGG
CTATTGTATGATTGTTCAGGTCTTTTGCCTTCAGCACCGAAGTTATACATCACATAA

Supplementary Material S3. Sequences of the Universal probes and primers used in ASQ and Amplifluor-like methods [31, 32].

ASQ molecular probes and allele-specific primers

Universal Probes (5′-3′):

Uni-ASQ-1: **FAM**-ccagctgaacggt***TCGACC***
Uni-ASQ-2: **HEX**-ccagctgaacggt***ACCTGC***
Uni-ASQ-Q: *accgttcagctgg*-**Q**

Allele-specific primers (5′-3′):

HvSAP8-ASQ-F1: ccagctgaacggt***TCGACC-***GAAGTTCTGCCATAGCCTCT**G**T
HvSAP8-ASQ-F2: ccagctgaacggt***ACCTGC-***GAAGTTCTGCCATAGCCTCT**C**T
HvSAP8-ASQ-R1: GAATCGACGGCCAGAGGAAAAT
Amplicon size = 78 bp

HvSAP16-ASQ-F1: ccagctgaacggt***TCGACC-***AGACGATGTTGGACAAGCTTC**T**C
HvSAP16-ASQ-F2: ccagctgaacggt***ACCTGC-***AGACGATGTTGGACAAGCTTC**A**C
HvSAP16-ASQ-R: GCACATTTGTACTGAGGATGGTAG
Amplicon size: 83 bp

Amplifluor-like molecular probes and allele-specific primers

Universal Probes (5′-3′):

Uni-1: **FAM**-AGCGATGCGTTCGAGCATCGC(T***-BHQ1)**gaaggt***GACCAAGTTCATGCT***
Uni-2: **HEX**-AGGACGCTGAGATGCGTCC(T***-BHQ1)**gaaggt***CGGAGTCAACGGATT***

Allele-specific primers (5′-3′):

HvSAP8-Amp-F1: gaaggt***GACCAAGTTCATGCT***-GAAGTTCTGCCATAGCCTCT**G**
HvSAP8-Amp-F2: gaaggt***CGGAGTCAACGGATT***-GAAGTTCTGCCATAGCCTCT**C**
HvSAP8-Amp-R: GAATCGACGGCCAGAGGAAAAT
Amplicon size = 78 bp

HvSAP16-Amp-F1: gaaggt***GACCAAGTTCATGCT***-AGACGATGTTGGACAAGCTTC**T**
HvSAP16-Amp-F2: gaaggt***CGGAGTCAACGGATT***-AGACGATGTTGGACAAGCTTC**A**
HvSAP16-Amp-R: GCACATTTGTACTGAGGATGGTAG
Amplicon size = 83 bp

Supplementary Material S4. Sequences of primers qPCR used in this study.

Name	Sequence (5′-3′)	Amplicon size (bp)
HvSAP8-qPCR-F	CCTCCTCTTTTGACAGCATCGTC	136
HvSAP8-qPCR-R	GGACCAGCGATGTCAGCAGG	
HvSAP16-qPCR-F	AGTTCCTGGGTGCAGAGAAGCA	208
HvSAP16-qPCR-R	GGAAGAACTACTGCTACTGCTGTT	
HvADP-qPCR-F	GCTCTCCAACAACATTGCCAAC	77
HvADP-qPCR-R	GAGACATCCAGCATCATTCATTCC	
HvGAPD-qPCR-F	GCCAAGACCCAGTAGAGC	78
HvGAPD-qPCR-R	CACATTTATTCCCATAGACAAAGG	
