# Supplementary material for: Expression of Specific Alleles of Zinc-Finger Transcription Factors, HvSAP8 and HvSAP16, and Corresponding SNP Markers, Are Associated with Drought Tolerance in Barley Populations
Source: Int J Mol Sci. 2021 Nov 10;22(22):12156. doi: 10.3390/ijms222212156 (PMC8617764; doi:10.3390/ijms222212156)
Supplement: Supplementary file 1 [file ijms-22-12156-s001.zip › SM-REF.pdf]

4. Vij, S.; Tyagi, A.K. Genome-wide analysis of the stress associated protein (SAP) gene family containing A20/AN1 zinc-finger(s) in rice and their phylogenetic relationship with *Arabidopsis*. *Mol. Genet. Genom.* **2006**, *276*, 565–575. https://doi.org/10.1007/s00438-006-0165-1.
10. Huang, J.; Wang, M.M.; Jiang, Y.; Bao, Y.M.; Huang, X.; Sun, H.; Xu, D.Q.; Lan, H.X.; Zhang, H.S. Expression analysis of rice A20/AN1-type zinc finger genes and characterization of *ZFP177* that contributes to temperature stress tolerance. *Gene* **2008**, *420*, 135–144. https://doi.org/10.1016/j.gene.2008.05.019.
11. Kanneganti, V.; Gupta, A.K. Overexpression of *OsiSAP8*, a member of stress associated protein (SAP) gene family of rice confers tolerance to salt, drought and cold stress in transgenic tobacco and rice. *Plant Mol. Biol.* **2008**, *66*, 445–462. https://doi.org/10.1007/s11103-007-9284-2.
12. Muthuramalingam, P.; Jeyasri, R.; Selvaraj, A.; Kalaiyarasi, D.; Aruni, W.; Pandian, S.T.K.; Ramesh, M. Global transcriptome analysis of novel stress associated protein (*SAP*) genes expression dynamism of combined abiotic stresses in *Oryza sativa* (L.). *J. Biomol. Struct. Dyn.* **2021**, *39*, 2106–2117. https://doi.org/10.1080/07391102.2020.1747548.
13. Martin, R.C.; Glover-Cutter, K.; Baldwin, J.C.; Dombrowski, J.E. Identification and characterization of a salt stress-inducible zinc finger protein from *Festuca arundinacea*. *BMC Res. Notes* **2012**, *5*, 66. https://doi.org/10.1186/1756-0500-5-66.
14. Li, W.; Zhang, L.; Soomro, F.; Guo, P.; Yuan, X.; Wang, Y. Foxtail millet stress associated protein gene *SiSAP4* enhances drought stress tolerance in transgenic *Arabidopsis*. *Int. J. Agric. Biol.* **2021**, *25*, 441–449. https://doi.org/10.17957/IJAB/15.1686.
15. Li, X.J.; Wu, Y.L.; Yang, B.P.; Wang, J.G.; Zhang, S.Z. Function analysis of sugarcane A20/AN1 zinc-finger protein gene *ShSAP1* in transgenic tobacco. *Crop Sci.* **2014**, *54*, 2724–2734. https://doi.org/10.2135/cropsci2014.01.0047.
16. Sreedharan, S.; Shekhawat, U.K.S.; Ganapathi, T.R. *MusaSAP1*, a A20/AN1 zinc finger gene from banana functions as a positive regulator in different stress responses. *Plant Mol. Biol.* **2012**, *80*, 503–517. https://doi.org/10.1007/s11103-012-9964-4.
17. Xu, Q.F.; Mao, X.G.; Wang, Y.X.; Wang, J.Y.; Xi, Y.J.; Jing, R.L. A wheat gene *TaSAP17-D* encoding an AN1/AN1 zinc finger protein improves salt stress tolerance in transgenic *Arabidopsis*. *J. Integr. Agric.* **2018**, *17*, 507–516. https://doi.org/10.1016/S2095-3119(17)61681-2.
18. He, X.; Xie, S.; Xie, P.; Yao, M.; Liu, W.; Qin, L.; Liu, Z.; Zheng, M.; Liu, H.; Guan, M.; et al. Genome-wide identification of stress-associated proteins (SAP) with A20/AN1 zinc finger domains associated with abiotic stresses responses in *Brassica napus*. *Environ. Exp. Bot.* **2019**, *165*, 108–119. https://doi.org/10.1016/j.envexpbot.2019.05.007.
19. Gao, W.; Long, L.; Tian, X.; Jin, J.; Liu, H.; Zhang, H.; Xu, F.; Song, C. Genome-wide identification and expression analysis of stress-associated proteins (SAPs) containing A20/AN1 zinc finger in cotton. *Mol. Genet. Genom.* **2016**, *291*, 2199–2213. https://doi.org/10.1007/s00438-016-1252-6.
20. Zhao, X.; Wang, R.; Zhang, Y.; Li, Y.; Yue, Y.; Zhou, T.; Wang, C. Comprehensive analysis of the stress associated protein (SAP) gene family in *Tamarix hispida* and the function of *ThSAP6* in salt tolerance. *Plant Physiol. Biochem.* **2021**, *165*, 1–9. https://doi.org/10.1016/j.plaphy.2021.05.016.
21. Wang, Z.; Kuang, J.; Han, B.; Chen, S.; Liu, A. Genomic characterization and expression profiles of stress-associated proteins (SAPs) in castor bean (*Ricinus communis*). *Plant Divers.* **2021**, *43*, 152–162. https://doi.org/10.1016/j.pld.2020.07.010.
22. Zhou, Y.; Zeng, L.; Chen, R.; Wang, Y.; Song, J. Genome-wide identification and characterization of stress-associated protein (SAP) gene family encoding A20/AN1 zinc-finger proteins in *Medicago truncatula*. *Arch. Biol. Sci.* **2018**, *70*, 87–98. https://doi.org/10.2298/ABS170529028Z.
45. Lai, W.; Zhou, Y.; Pan, R.; Liao, L.; He, J.; Liu, H.; Yang, Y.; Liu, S. Identification and expression analysis of stress-associated proteins (SAPs) containing A20/AN1 zinc finger in cucumber. *Plants* **2020**, *9*, 400. https://doi.org/10.3390/plants9030400.
46. Solanke, A.U.; Sharma, M.K.; Tyagi, A.K.; Sharma, A.K. Characterization and phylogenetic analysis of environmental stress-responsive *SAP* gene family encoding A20/AN1 zinc finger proteins in tomato. *Mol. Genet. Genom.* **2009**, *282*, 153–164. https://doi.org/10.1007/s00438-009-0455-5.
47. Zhang, X.Z.; Zheng, W.J.; Cao, X.Y.; Cui, X.Y.; Zhao, S.P.; Yu, T.F.; Chen, J.; Zhou, Y.B.; Chen, M.; Chai, S.C.; et al. Genomic analysis of stress associated proteins in soybean and the role of *GmSAP16* in abiotic stress responses in *Arabidopsis* and soybean. *Front. Plant Sci.* **2019**, *10*, 1453. https://doi.org/10.3389/fpls.2019.01453.
48. Lloret, A.; Conejero, A.; Leida, C.; Petri, C.; Gil-Muñoz, F.; Burgos, L.; Badenes, M.L.; Ríos, G. Dual regulation of water retention and cell growth by a *stress-associated protein* (*SAP*) gene in *Prunus*. *Sci. Rep.* **2017**, *7*, 332. https://doi.org/10.1038/s41598-017-00471-7.
